# Characterization of contrast-mediated collinear interactions in the human visual system

**DOI:** 10.1038/s41598-025-94361-y

**Published:** 2025-04-07

**Authors:** Luca Battaglini, Giulio Contemori, Gianluca Campana, Marco Bertamini, Gianluca Ruffato, Marcello Maniglia

**Affiliations:** 1 Department of General Psychology, Università di Padova, Padua, Italy; 2 Department of Physics, Università di Padova, Padua, Italy; 3 Department of Psychology, University of California Riverside, Riverside, CA USA

**Keywords:** Contextual modulation, Collinear facilitation, Contrast sensitivity, Human behaviour, Sensory processing

## Abstract

Collinear modulation represents a fundamental building block of our perceptual world, and its study has enhanced our understanding of contrast sensitivity, spatial integration, and neural plasticity in the visual system. In this phenomenon, the visibility of a target is influenced by collinear elements. Four key factors modulate this effect: the distance between target and flankers, the contrast of the flankers, retinal eccentricity, and spatial frequency. Each of these elements affects collinear modulation, increasing or reducing the target’s visibility. Short target-to-flankers separations decrease contrast sensitivity, while larger separations increase it. However, this pattern holds for high contrast flankers, whereas low contrast flankers increase contrast sensitivity at short separations, while the effect is weaker or absent at large separations. Moreover, eccentricity appears to increase the spatial extent of inhibition. Across three experiments, we systematically varied these key factors. In Experiments 1 and 2, we manipulated spatial frequency, flankers’ separation, and flankers’ contrast in foveal vision. In Experiment 3, we varied flankers’ separation and flankers’ contrast in peripheral vision. The results reveal a complex pattern that may help reconcile previously conflicting findings in both healthy participants and clinical populations, offering an updated framework for the study of neural plasticity in the visual system.

**Supplementary Information:**

The online version contains supplementary material available at 10.1038/s41598-025-94361-y.

## Introduction

Contrast sensitivity (CS), the ability to discern differences in luminance, constitutes the foundation of our visual experience. A substantial body of literature highlights its role in human perception and its correlation with the ability to perform essential daily life activities, such as reading and recognizing objects^1^.

While visual acuity (VA) has long been considered the standard measure of visual function, it primarily focuses on the resolution of letters displayed at high contrasts. However, in common visual pathologies such as macular degeneration (MD), best-corrected VA often remains intact at early stages, thus providing limited insight into disease progression until the later stages^2^. Both health professionals and researchers acknowledge that VA tests do not accurately predict the visual abilities required for daily activities^3^, while evidence suggests that loss of CS, in clinical population, correlates more strongly to reduction of quality of life and negative psychological consequences than reduction of VA^4,5^, suggesting that sometime the loss of CS can be more psychologically disturbing than the loss of visual acuity.

One effective approach to visual rehabilitation uses CS effects, specifically a contextual modulation phenomenon known as collinear modulation (including collinear facilitation and inhibition, e.g^6–13^). It has been proposed that the interactions at the foundation of collinear modulation enable detection of edges and texture borders (see^14^).

In this paper, we combined replications with novel experiments to systematically characterize collinear modulation in foveal and peripheral vision. A better understanding of this fundamental mechanism of visual integration can provide insights into early stages of visual processing and guide the interpretation of clinical data in the context of cortical adaptation to visual impairment.

Typically, collinear modulation studies feature a central target, usually a Gabor patch, flanked by iso-oriented, collinear Gabor patches, in a lateral masking configuration. A number of studies over the last few decades showed that this configuration can lead to an increase or a decrease in contrast sensitivity for the central target, a phenomenon which has been linked to the horizontal connections between units in hypercolumns sharing the same orientation and spatial frequency tuning^15,16^. As such, changes in characteristics of collinear modulation following training or visual pathologies have been considered evidence of plasticity in early stage of visual processing^7,8,17^.

Four key aspects seem to contribute to collinear modulation: target-to- flankers distance, the contrast of the target and the flankers, retinal eccentricity, and spatial frequency^6^.

*Target-to- flankers distance*: early studies in foveal vision showed that the presence of flanking elements placed at a distance larger than twice the wavelength (λ) of the target produces an enhancement (facilitation) of the target’s CS^8,18^. This facilitatory effect has its peak at 3λ, with one animal electrophysiology study showing increase in V1 neuron response to a central target when flankers are presented at 3λ^19^, and could be observed up to 8–12λ in some individuals^20^. On the other hand, when flankers are placed at a distance closer than 2λ, CS for the central target is reduced^8,18^.

*Flankers and target contrast*: electrophysiological and psychophysical studies have shown that contrast affects collinear modulation^19,21^. Specifically, high-contrast flankers inhibit target detection at high target contrast but facilitate it when the target contrast is low. Zenger and Sagi^21^ proposed a model based on psychophysical data that predicts a distance-dependent multiplicative effect on contrast, specifically that the target vs. contrast (TvC) curve shifts to the right (higher target contrast required for detection) as the target-to-flankers separation increases. This model describes contextual modulation as being dependent on the contrast of the flankers relative to the contrast of the target. When the target-to-flanker separation is small (2λ) and the contrast of the flankers is approximately three times the individual threshold measured under baseline conditions (i.e., without flankers), contrast sensitivity improves. However, if the contrast of the flankers exceeds 10 times the contrast of the baseline contrast (e.g., high contrast flankers and low contrast target), target perception is inhibited. At larger separations (4λ), maximal facilitation occurs when the flankers’ contrast is about ten times higher than the baseline contrast, while inhibition occurs when the flankers’ contrast is more than sixty times the baseline contrast. While the specific numerical predictions may not generalize due to differences in stimulation parameters like presentation duration and ISI, the key aspect of Zenger and Sagi’s model is the distance-dependent multiplicative effect of flanker contrast on target detection. Electrophysiology evidence shows that neurons’ response to a near-threshold contrast Gabor patch placed within their receptive fields is modulated by the presence of high-contrast flankers placed outside their receptive fields^19^. Specifically, the cells’ response increased in the presence of flankers placed at a separation of 3λ; however, when the target had higher contrast values, exceeding 1/4 of the flankers contrast, the strength of the neural response was reduced (suppressed). Further psychophysical studies by Chen and Tyler^22,23^ support the idea that low contrast flankers positioned at short distance from the target facilitated target detection, consistent with previous studies^8,18,21,24^, but the strength of collinear facilitation dramatically decreased when the contrast of the target increases.

*Retinal eccentricity*: collinear facilitation has also been observed in parafovea and in peripheral vision (configuration eccentricity of 4° or more). Giorgi and colleagues^25^ found weak facilitation with a temporal forced-choice paradigm. Facilitation was also found by Maniglia and colleagues^6,11,26^ with a facilitatory peak found at 6–8λ. This larger inhibitory region in peripheral vision has been linked to cortical magnification, according to which the size of the receptive fields of neurons processing peripheral visual field increases with eccentricity. However, Shani and Sagi^27^ reported that collinear modulation seems to be less stable among participants (i.e., not all participants exhibited collinear modulation) as the configuration is moved further into the periphery.

*Spatial frequency*: studies in fovea and periphery show contrasting results: Polat, testing participants in foveal vision, showed maximal facilitation for high spatial frequencies and minimal for low spatial frequencies^28^. The author interpreted these results in terms of propagation speed and integration time between the signals from the flankers and the target. Facilitation was reduced when the flankers were farther away in retinal space, as it is the case for low spatial frequencies in the lateral masking paradigm, in which separations are relative to the wavelength (λ) of the stimuli. Conversely, Maniglia and colleagues found that participants tested in the periphery (4° of eccentricity) exhibited the opposite, with largest facilitation at lower spatial frequencies^6^. This finding is consistent with the known spatial frequency tuning of the periphery of the visual field^29^.

Clinical relevance:

Although this study does not directly test patients, it aims to better inform clinical research that employs the lateral masking paradigm as a tool to train or assess cortical plasticity in clinical populations. By quantitatively measuring the spatial range and intensity of facilitation and inhibition, the paradigm has proven valuable for identifying functional alterations in individuals with visual impairments, such as macular degeneration^9,17,30^ and amblyopia^7,31^.

Beyond its diagnostic utility, the lateral masking paradigm enables longitudinal tracking of cortical changes during visual rehabilitation or neuroplasticity interventions, such as perceptual learning and brain stimulation^32–34^. However, in the context of collinear modulation measured with lateral masking, patients with low contrast sensitivity exhibit two simultaneous effects: elevated contrast detection thresholds for isolated targets and reduced perceived contrast of the flankers. This conflation, which may account for some of the discrepancies between healthy participants and the clinical literature, has often been underestimated.

Zenger and Sagi^21^ describe contextual modulation as being dependent on the contrast of the flankers relative to the contrast of the target. Similarly, Maniglia and colleagues showed that the degree of facilitation and inhibition can be modulated by contrast adaptation of the mask, highlighting the impact of perceived contrast on contextual modulation^35^. Consequently, facilitation and inhibition effects in clinical populations are necessarily altered—not solely due to cortical adaptations from the pathology, but more fundamentally because of reduced contrast sensitivity.

A better understanding of collinear modulation can help with characterizing mechanisms of visual integration in both healthy and clinical population, possibly offering insights into mechanisms of cortical reorganization. To better characterize this early-stage phenomenon, we conducted a series of psychophysical experiments, parts replications and parts novel studies, to explore the relationship between four key factors in collinear modulation: spatial frequency, eccentricity, target-to-flankers separation and flanker contrast.

Specifically, in the first two experiments, we manipulated spatial frequencies, flankers contrast and target-to-flankers separation, comparing our results with estimation from the model of Zenger and Sagi^21^.

Lastly, in a third experiment, we tested different target-to-flankers separation and flankers contrast in peripheral vision, testing, for the first time, the Zenger and Sagi^21^ model in the periphery of the visual field.

## Method

### Participants

80 participants took part in this study. 20 participants (4 males and 16 females, mean age = 21.35, SD = 1.63), participated in Experiment 1; 20 participants (9 males and 11 females, mean age = 23.25, SD = 5.55) took part in Experiment 2, and 40 participants (14 males and 26 females, mean age = 22.45, SD = 2.96) were included in Experiment 3. Participants were recruited without prior knowledge of the study’s purpose and voluntarily participated. All participants had normal or corrected-to-normal vision. All the participants gave a written informed consent to participate in the study. The study adhered to the ethical standards outlined in the Declaration of Helsinki (1964), and the protocol (no. 4732) received approval from the Ethics Committee for Psychological Research of the University of Padova.

### Apparatus

Stimuli were displayed on a 20” CRT monitor (Philips 202P) with a resolution of 1600 × 1200 and a refresh rate of 85Hz. The monitor was connected to an ASUS P8H61-M LX2 computer, powered by an Intel Core i5 3450 processor, running Windows 7 Pro 64bit SPI operating system. The average luminance of the monitor, corresponding to the middle gray used as background colour throughout the study, was set to 44.8 cd/m^2^. Each pixel subtended 1.5 arcmin. A ColorCal2 colorimeter (Cambridge Research Systems) was used to calibrate the monitor, and gamma correction was achieved using a 12-bit lookup table (LUT) to ensure that luminance was a linear function of the digital image representation. An NVIDIA GeForce GT 220 graphics card, in conjunction with a Bits + + system (Cambridge Research System), facilitated a luminance resolution of 12-bit (4096 levels of grayscale). MATLAB (Mathworks, Inc., version 7.8.0.347) and Psychophysics Toolbox^36,37^ were utilized to program the experiments. The monitor was positioned at 70 cm from the participants for all the Experiment except for Experiment 2, in which the monitor was positioned at 114 cm. This adjustment was necessary to accommodate the accurate display of 12 cycles per degree (cpd) stimuli used in Experiment 2. All the experiments were conducted in a dark room. A chin rest was employed to stabilize participants’ heads, ensuring consistent viewing conditions. Participants were instructed to maintain their focus on the fixation point at the centre of the screen without shifting their gaze. Vision was binocular.

### Stimuli

The visual stimuli consisted of Gabor patches, which were comprised of a cosinusoidal carrier enveloped by a stationary Gaussian, as defined by Eq. 1.

1 $$\:G\:\left(x,\:y\right)=cos\:\left(\frac{2\pi\:}{\lambda\:}\:x+\:\phi\:\right){e}^{(-\frac{{x}^{2}+{y}^{2}}{{\sigma\:}^{2}})}$$

**Fig. 1:**
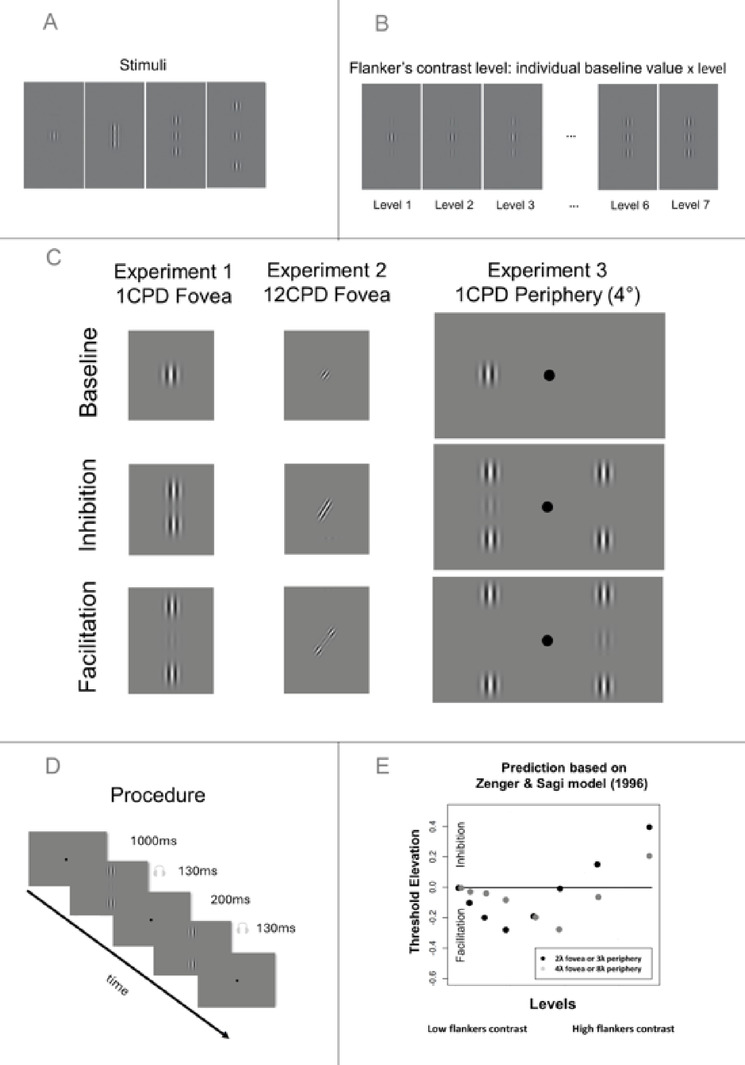
(**A**) Representation of the stimuli used in the lateral masking paradigm to study lateral interactions. (**B**) Representation of the flankers with varying levels of contrast. The contrast of the flankers was calculated by measuring the contrast threshold for an isolated Gabor patch. The threshold value is then multiplied by selected values to test the model of Zenger and Sagi. (**C**) Representation of the stimuli used in the experiments. (**D**) Representation of the experimental procedure (2 interval forced choice, 2IFC). (**E**) Expected results according to the model of Zenger and Sagi.

Here, λ represents the sinusoidal wavelength, φ represents the phase, and σ represents the standard deviation of the luminance Gaussian envelope (σ = 1 deg) in the (x, y) space of the image. In all experimental conditions, the Gabor’s standard deviation matched the carrier period (σ = λ), with fixed spatial phase (φ = 0) and a spatial frequency of 1 cpd, except for Experiment 2 in which the spatial frequency was 12 cpd. Examples of these stimuli are presented in Fig. 1. The target was presented either alone (baseline) or in the classic collinear configuration (see Fig. 1A). In Experiment 1, the configuration was presented vertically and in the centre of the screen (collinear configuration). In Experiment 2, the configuration was oblique, with the elements rotated 45 degrees to the right to maintain collinearity across elements. In Experiment 3, the stimuli were placed at an eccentricity of 4 degrees, with both target and flankers collinear and vertically oriented. In Experiment 3, flankers were presented on both sides in every trial to prevent participants from using flankers as cues to reduce spatial uncertainty (see Fig. 1C). The center-to-center target-to-flanker separations varied across experiments and was defined as multiples of the Gabor’s carrier wavelength unit (λ). In Experiment 1, flankers were positioned at 2λ and at 4λ, distances at which high contrast flankers have inhibitory and facilitatory effects, respectively^18,21^. To generalize the results of Experiment 1 to different parameters, in Experiment 2 we used stimuli with a spatial frequency of 12 cpd, with flankers placed at either inhibitory (1.5λ, partially overlapped with the target) or facilitatory (3λ) separations. In Experiment 3, collinear flankers were placed at 3λ within the peripheral region of collinear inhibition^27^ and at 8λ to elicit facilitation^11,25^. These variations in target-to-flanker separations were informed by previous research showing the influence of spatial arrangement on visual perception and performance.

Flanker contrast was varied on a logarithmic scale composed of seven levels defined as multiples of each participant’s baseline. Specifically, these levels were: [individual baseline value, i.e., the threshold contrast obtained without flankers] × 0.56, 1.24, 2.71. 5.96, 13.08, 28.73, 63.09 for Experiment 1; [individual baseline value] x 0.1, 0.22, 0.48, 1, 2.15, 4.64 and 10 in experiment 2; and [individual baseline value] x 0.12, 0.27, 0.56, 1.24, 2.71. 5.96, 13.08, 28.73, 63.09 in Experiment 3. These values were chosen because they allowed for sampling both the ‘facilitatory’ and ‘inhibitory’ parts of the model by Zenger and Sagi^21^; see Fig. 10b).

### General experimental procedure

Participants completed eight experimental blocks. The contrast of the flankers was varied between blocks. Block order was counterbalanced across participants, except for the first block where flankers contrast was always set to 0 (individual baseline condition). A 2-IFC task was used in each block. A trial consisted of a 1000ms fixation period, followed by two sequential intervals (130ms each) whose onset was indicated, each time, by a tone presentation (pure tone at 400 Hz). The two sequential intervals were separated by an inter stimulus interval (ISI) of 200ms, in which a central fixation dot was present. Only one of the two intervals contained the target, randomised on a trial basis, while both intervals contained the flankers (Fig. 1). Participants had to report which interval contained the target by pressing a key on a computer keyboard. Auditory feedback informed the participant on the response accuracy. In all experiments, the contrast of the target was set to start at 10% contrast and was varied according to a 3:1 staircase^38^, increasing the contrast by 0.1 log units for every inaccurate response and decreasing it by the same amount after 3 consecutive correct responses. The staircase procedure ended after 120 trials or 14 reversals. Target detection thresholds, equivalent to 79% of correct responses, were computed averaging the contrast values of the last 6 reversals. Apart from the target contrast, all stimulus parameters were kept constant within one block.

In Experiment 1 and 2 the target was presented in the fovea. In Experiment 3 the target was presented at 4° in periphery, either to the left or to the right of the central fixation dot, while flankers were presented on both sides at the same time.

### Data analysis

Analyses were performed in R. We analyzed threshold elevation (TE) as defined by Eq. 2 ^31^. Positive values indicate that the threshold measured in the flanker condition is higher (worse performance, inhibitory effect of the flankers) than baseline. Negative values indicate better performance compared to the baseline, thus suggesting facilitatory effect of flankers.

2 $$\:{\text{Threshold\:Elevation}=\text{LOG}}_{10}\frac{collinear\:configuration}{baseline}$$

We analyzed TE by fitting a linear mixed model with two factors, “Flankers contrast” and “Lambda”. To control for the within-subjects correlation typical of repeated measures, we also included an individual random intercept and individual random slope. Mixed models were estimated with a Restricted Maximum Likelihood procedure (REML) with the function “lmer()” from the “lme4” package^39^. Next, to assess the fixed effects we used a type III Wald-test implemented via the Anova() function from the CAR package. Orthogonal polynomial contrasts were applied to the flanker contrasts levels to investigate potential facilitation or inhibition patterns, adjusting for false discovery rate (FDR) across 6 tests. These contrasts included quadratic and higher degree terms, serving as hierarchical tests to evaluate whether a non-linear component improves model fit beyond linear effects. To pinpoint the location of peaks or dips in TE, we compared each flanker contrast against a null hypothesis (TE = 0) within each flanker distance condition using 7 separate contrasts. Confidence intervals were adjusted with Bonferroni correction for 7 estimates at a 95% confidence level. Furthermore, p-values from t-tests were adjusted using the FDR method for 7 tests. The orthogonal polynomial contrasts and comparisons with baseline were computed using the “emmeans()” function from the “emmeans” package. Thresholds that were more than three standard deviations from the average were considered outliers and the corresponding TEs were removed from analysis. In Experiment 3 we collected 20 participants but an error in the task script led to wrong multiplier levels for the flankers (0.123 and 0.271 instead of 1.24 and 2.71). We collected a further sample of 20 participants with the correct multiplier values. We subsequently decided to analyze the two groups together to characterize the effects of very low flankers contrast values. It is noteworthy that the analytical approach with mixed models is very robust and allows the study of experimental conditions with different numbers of participants/trials and within/between effects within the same model^40^.

## Results

### Exp 1: foveal collinear modulation for different flanker contrasts at low Spatial frequency

**Fig. 2:**
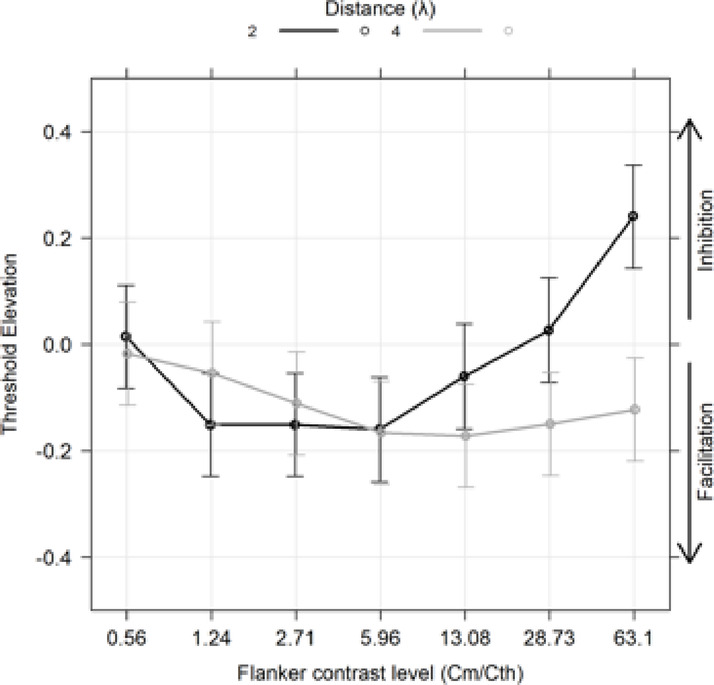
Results obtained in Experiment 1. The spatial frequency of the Gabor patches was 1 CPD. For a target-flankers distance of 2λ, facilitation was observed for low levels of flanker contrast, as predicted by the Zenger & Sagi model^21^. However, as flanker contrast increases, facilitation transitions to inhibition. For a target-flankers distance of 4λ, facilitation increases with flanker contrast, apparently reaching a plateau. In the figure, Cm represents the contrast of the flankers, while Cth indicates the contrast threshold in the baseline condition (i.e., the threshold for detecting the target stimulus without flankers).

Experiment 1 examined the effect of target-to-flanker distances (2λ and 4λ) on visual performance with varying flanker contrasts (Fig. 2). The TE was modeled using a linear mixed model, with flanker contrast as a 7-level ordered factor and distance (λ) as a 2-level categorical factor. According to the lme4 package notation in R: $\:TE\:\sim\:flanker\:contrast\:level\:\text{*}\:\text{f}\text{l}\text{a}\text{n}\text{k}\text{e}\text{r}\:\text{d}\text{i}\text{s}\text{t}\text{a}\text{n}\text{c}\text{e}\:\left({\uplambda\:}\right)\:+\:\left(1\:\right|\:subj)$. The type III Wald test on the model fit revealed significant effects. Specifically, there were main effects of flanker contrast (χ²(6) = 49.978, *p* < 0.001) and distance (λ) (χ²(1) = 14.686, *p* < 0.001), as well as a significant interaction between flanker contrast and distance (χ²(6) = 48.967, *p* < 0.001). These results indicate that there was a clear effect of flanker contrast, but that this effect differed for the 2λ and 4λ conditions. Additionally, orthogonal polynomial contrasts revealed significant linear and quadratic effects for the 2λ condition (linear: t(281) = 5.525, *p* < 0.001; quadratic: t(281) = 7.203, *p* < 0.001), whereas for the 4λ condition, only a significant linear effect was found (linear: t(281) = -2.800, *p* = 0.0331). These results indicated a non-linear effect for the flanker contrast relative to the 2λ TE as evidenced by the black U-shaped curve in Fig. 2. Comparisons between flanker contrast and the null hypothesis (zero TE) at the target-to-flanker distance of 2λ are presented in Table 1. levels 1.24, 2.71, and 5.96 exhibited significantly negative marginal means, while at level 63.1, the marginal mean was significantly positive. These results indicate that at 2λ, low flanker contrast facilitated performance, with a switch to inhibition at high contrast levels, consistent with previous findings^18,21^. Comparisons between flanker contrast and the null hypothesis at 4λ are presented in Table 2. at a level 2.71, the marginal mean was − 0.111, significantly different from zero (*p* = 0.036). At all levels beyond 1.24, the marginal means were significantly lower than zero.

**Table 1:** Estimated marginal means for each flanker contrast levels at 2λ.

Level	Emmean	SE	df	Lower CL	Upper CL	t. ratio	*p*. value
0.56	0.014	0.049	87.6	− 0.121	0.149	0.293	0.77
1.24	− 0.15	0.049	87.6	− 0.287	− 0.015	− 3.071	0.005*
2.71	− 0.15	0.049	87.6	− 0.287	− 0.015	− 3.076	0.005*
5.96	− 0.16	0.05	92.3	− 0.298	− 0.022	− 3.198	0.005*
13.08	− 0.06	0.05	92.3	− 0.198	0.077	− 1.204	0.324
28.73	0.026	0.05	92.3	− 0.111	0.164	0.528	0.698
63.1	0.240	0.049	87.6	0.105	0.376	4.890	< 0.0001*

**Table 2:** Estimated marginal means for each flanker contrast levels at 4λ.

Level	Emmean	SE	df	Lower CL	Upper CL	t. ratio	*p*. value
0.56	− 0.017	0.049	87.6	− 0.153	0.118	− 0.356	0.722
1.24	− 0.054	0.049	87.6	− 0.190	0.081	− 1.102	0.319
2.71	− 0.111	0.049	87.6	− 0.247	0.024	− 2.261	0.036*
5.96	− 0.166	0.049	87.6	− 0.302	− 0.03	− 3.376	0.003*
13.08	− 0.172	0.049	87.6	− 0.308	− 0.036	− 3.499	0.003*
28.73	− 0.149	0.049	87.6	− 0.285	− 0.014	− 3.044	0.007*
63.1	− 0.122	0.049	87.6	− 0.258	0.012	− 2.492	0.025*

These results indicate that flankers placed at 4λ elicited facilitatory effects, consistent with previous findings^18,21^.

### Exp 2: foveal collinear modulation for different flanker contrasts and high Spatial frequency

**Fig. 3:**
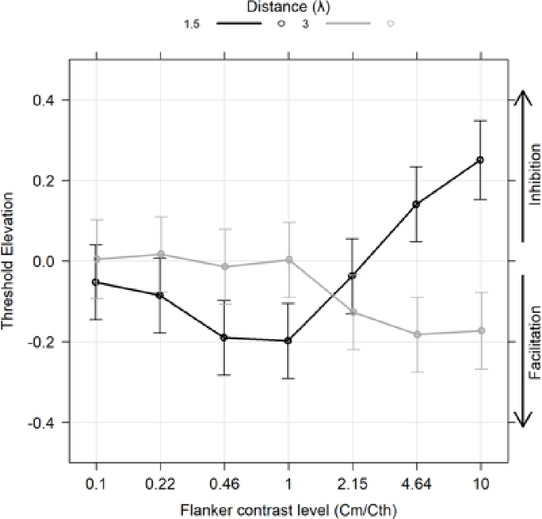
Results obtained in Experiment 2. The spatial frequency of the Gabor patches was 12 CPD. For a target-flankers distance of 1.5λ, facilitation was observed for low levels of flanker contrast. These results extend the predictions of the Zenger & Sagi^21^ model to a different configuration (oblique) and spatial frequency. As in the previous experiment, as flanker contrast increases, facilitation transitions to inhibition. For a target-flankers distance of 3λ, facilitation increases with flanker contrast, apparently reaching a plateau. In the figure, Cm represents the contrast of the flankers, while Cth indicates the contrast threshold in the baseline condition (i.e., the threshold for detecting the target stimulus without flankers).

Experiment 2 investigated the impact of inhibitory (1.5λ) and facilitatory (3λ) target-to-flanker distances on contrast detection for different flanker contrasts at 12 cpd in the fovea (Fig. 3). The TE was modeled using a linear mixed-effects model, with flanker contrast (level) as a 7-level ordered factor and distance (λ) as a 2-level categorical factor. According to the lme4 package notation in R: $\:TE\:\sim\:flanker\:contrast\:level\:\text{*}\:\text{f}\text{l}\text{a}\text{n}\text{k}\text{e}\text{r}\:\text{d}\text{i}\text{s}\text{t}\text{a}\text{n}\text{c}\text{e}\:\left({\uplambda\:}\right)\:+\:\left(1\:\right|\:subj)$. The type III Wald test on the model fit revealed significant effects, specifically the main effect of flanker contrast (χ²(6) = 15.127, *p* = 0.019), the main effect of flankers distance (χ²(1) = 3.188, *p* = 0.074), and their interaction (χ²(6) = 89.222, *p* < 0.001). These results indicate significant effects of flanker contrast and its interaction with distance, with a marginal effect of distance alone. Additionally, orthogonal polynomial contrasts revealed significant linear and quadratic effects for the 1.5λ condition (linear: t(242) = 6.323, *p* < 0.0001; quadratic: t(242) = 5.956, *p* < 0.0001), and a significant linear effect for the 3λ condition (linear: t(243) = -4.322, *p* = 0.001). Comparisons between flanker contrast and the null hypothesis (zero TE) at 1.5λ are shown in Table 3. At levels 0.46 and 1, the estimated marginal means were significantly lower than zero, while at levels 4.64 and 10, the estimated marginal means were significantly higher than zero. At all the other levels was not significantly different from zero. Table 3 summarizes the results for different levels at 1.5λ.

**Table 3:** Estimated marginal means for the flanker contrast levels at 1.5λ.

Level	Emmean	SE	df	Lower CL	Upper CL	t. ratio	*p*. value
0.1	− 0.052	0.047	224	− 0.180	0.076	− 1.108	0.314
0.22	− 0.085	0.047	224	− 0.213	0.043	− 1.810	0.1
0.46	− 0.190	0.047	224	− 0.318	− 0.062	− 4.028	< 0.001*
1	− 0.198	0.047	224	− 0.326	− 0.070	− 4.196	< 0.001*
2.15	− 0.038	0.047	224	− 0.166	0.091	− 0.796	0.427
4.64	0.141	0.047	224	0.013	0.269	2.985	0.006*
10	0.251	0.050	232	0.116	0.385	5.059	< 0.0001*

At 3λ, levels 2.15, 4.64, and 10 showed estimated marginal means significantly below zero. Table 4 summarizes the results for this separation.

**Table 4:** Estimated marginal means for each the flanker contrast levels at 3λ.

Level	Emmean	SE	df	Lower CL	Upper CL	t. ratio	*p*. value
0.1	0.005	0.050	232	− 0.130	0.139	0.096	0.947
0.22	0.017	0.047	224	− 0.111	0.145	0.352	0.947
0.46	− 0.014	0.047	224	− 0.142	0.114	− 0.290	0.947
1	0.003	0.047	224	− 0.125	0.131	0.067	0.947
2.15	− 0.127	0.047	223	− 0.255	0.002	− 2.679	0.019*
4.64	− 0.183	0.047	224	− 0.311	− 0.055	− 3.871	0.001*
10	− 0.173	0.048	228	− 0.304	− 0.042	− 3.580	0.001*

These results suggest that at 3λ, higher flanker contrast levels generally lead to collinear facilitation, whereas 1.5λ elicits a mix of facilitation and inhibition, with significant facilitation at intermediate flankers’ contrast levels and inhibition at higher flankers’ contrast levels.

### Exp 3: peripheral collinear modulation for different flanker contrasts

**Fig. 4:**
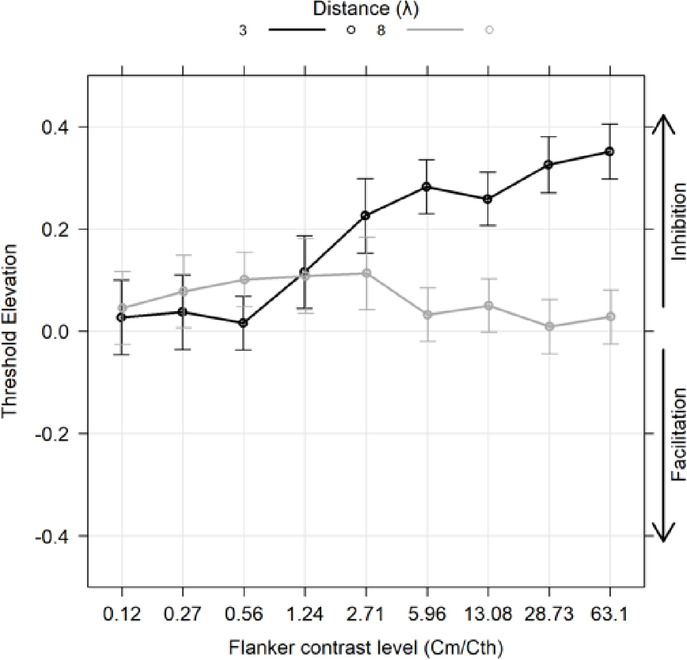
Results obtained in Experiment 3 with peripheral stimuli. The spatial frequency of the Gabors was 1 CPD. For a target-flankers distance of 3λ, inhibition was observed for all levels of flanker contrast, except for the lowest level, where flanker contrast seemed to have no effect. For a target-flankers distance of 8λ, flanker contrast appeared to have no effect (or produced only weak inhibition). In the figure, Cm represents the contrast of the flankers, while Cth indicates the contrast threshold in the baseline condition (i.e., the threshold for detecting the target stimulus without flankers).

In Experiment 3 we tested the effect of inhibitory (3λ) and facilitatory (8λ) separations on contrast detection for different flanker contrasts at an eccentricity of 4 deg (Fig. 4). The TE was modeled using a linear mixed-effects, with flanker contrast as a 7-level ordered factor, distance (λ) as a 2-level categorical factor, and orientation as a 2-level categorical factor. According to the lme4 package notation in R: $\:TE\:\sim\:flanker\:contrast\:level\:*\:flanker\:distance\:\left(\lambda\:\right)\:+\:\left(1\:\right|\:subj)$. The type III Wald test on the model fit revealed a significant main effect of flanker contrast (χ²(8) = 63.541, *p* < 0.001), a significant main effect of flanker distance (χ²(1) = 76.575, *p* < 0.001), and the interaction between the two (χ²(8) = 146.392, *p* < 0.001). We also found that the cubic polynomial contrasts for 3λ was significant (cubic: t(494) = -2.423, *p* = 0.0472), while for lambda 8 no significant polynomial contrasts were observed. Comparisons between flanker contrast and the null hypothesis for a separation of 3λ condition with iso-orientation show significantly higher than zero means for flanker contrast level of 1.24 and all those above. Table 5 summarizes the results for different levels at 3λ.

**Table 5:** Estimated marginal means for each of the 7 flanker contrast levels at 3λ.

Level	Emmean	SE	df	Lower CL	Upper CL	t. ratio	*p*. value
0.12	0.027	0.037	487	− 0.076	0.131	0.730	0.524
0.27	0.037	0.037	487	− 0.066	0.141	1.007	0.404
0.56	0.016	0.027	341	− 0.058	0.09	0.597	0.551
1.24	0.116	0.036	480	0.015	0.217	3.189	0.002*
2.71	0.226	0.037	487	0.123	0.33	6.085	< 0.0001*
5.96	0.282	0.027	347	0.207	0.358	10.481	< 0.0001*
13.08	0.259	0.027	341	0.184	0.333	9.706	< 0.0001*
28.73	0.326	0.028	368	0.248	0.404	11.685	< 0.0001*
63.1	0.351	0.027	354	0.275	0.428	12.897	< 0.0001*

Comparisons between flanker contrast and the null hypothesis of zero TE for the target-to-flanker distance of 8λ condition with iso-orientation at levels 0.56, 1.24, and 2.71, estimated marginal means were significantly positive (*p* < 0.05). Table 6 summarizes the results for different levels at 8λ.

**Table 6:** Estimated marginal means for each of the 7 flanker contrast levels at 8λ.

Level	Emmean	SE	df	Lower CL	Upper CL	t. ratio	*p*. value
0.12	0.046	0.036	480	− 0.055	0.147	1.259	0.285
0.27	0.078	0.036	480	− 0.023	0.179	2.142	0.074
0.56	0.101	0.027	347	0.026	0.177	3.758	0.002*
1.24	0.108	0.037	487	0.005	0.211	2.908	0.011*
2.71	0.113	0.036	480	0.012	0.215	3.128	0.008*
5.96	0.033	0.027	341	− 0.042	0.107	1.224	0.285
13.08	0.050	0.027	341	− 0.024	0.125	1.892	0.107
28.73	0.009	0.027	347	− 0.066	0.084	0.339	0.735
63.1	0.028	0.027	347	− 0.047	0.103	1.047	0.333

## Discussion

Early mechanisms of excitation and inhibition within the visual cortex shape the way we perceive the world. The interaction of horizontal inhibition and feedforward activation constitutes the building blocks of our visual experience. In a seminal paper, Polat and Sagi^18^ showed that the presence of flanking elements modulates the contrast sensitivity for a central target, with short separation leading to collinear inhibition, the reduction of contrast sensitivity, and larger separations leading to collinear facilitation, an increase in contrast sensitivity. Subsequent studies showed how several parameters, including the flankers contrast^21^, the spatial frequency of the stimuli^6,28^and the eccentricity at which the configuration is presented^11,41^ further modulate this effect. Here we systematically manipulated these four key elements, target-to-flankers separations, flanker contrast, and foveal vs. peripheral presentation to characterize collinear modulation across multiple factors.

Below we summarize the main findings.

*Foveal collinear modulation and comparison with Zenger and Sagi*^21^ *model*: In Experiment 1 and 2, in which the collinear configuration was presented in fovea, we observed clear facilitation at 1.5-2λ when the contrast of the flankers was less than 3 times the baseline. This effect did not seem to depend on spatial frequency or the global orientation of the configuration (Experiment 2). Zenger and Sagi^21^ tested a few observers, and used contrast, orientation, and phase of the flankers to build their model. Given individual variability and the limited number of participants tested by^21^, a direct comparison with our data could be misleading. However, we made a qualitative observation by plotting our data at 2λ on the same graph as the data obtained by^21^. This figure is in the supplementary materials. From our data, it emerges that, with respect to our data, the facilitation in the Zenger and Sagi model was larger, and the switching point from facilitation to inhibition is reached earlier (at lower flanker contrast). Overall, results from the foveal experiments provide an alternative interpretation to clinical results in patients with amblyopia and macular degeneration. Specifically, a study in patients with amblyopia showed larger suppression at every target-to-flankers separation compared to controls^7^. Moreover, the pattern of collinear modulation in amblyopic patients showed marked variability. Abnormalities in collinear facilitations increase as spatial frequencies increases and seem to depend on the type of amblyopia (strabismic, anisometropic, meridional amblyopia)^31^.

In macular degeneration (MD), a pathology that compromises central vision and forces patients to use their peripheral vision, collinear inhibition at short target-to-flanker separations was found to be significantly reduced when compared to age-matched participants tested at similar eccentricities^9,17^. The authors interpreted this as evidence of practice-induced cortical reorganization, similar to what Chung^42^ suggested to explain the shape of the crowding zone in the preferred retinal locus (PRL). Similarly, Contemori and colleagues^30^, measured collinear modulation in the PRL of patients with MD, showing clear facilitation even at 2λ, and up to 8λ, unlike eccentricity-matched controls, who showed the typical pattern of inhibition at 2λ and facilitation for larger target-to-flankers distances.

While both macular degeneration (MD) and amblyopia exhibit altered patterns of collinear modulation, these changes may stem from different underlying mechanisms. In MD, the abnormalities are likely related to retinal damage and the resulting limited retinal input, whereas in amblyopia, they are attributed to extensive cortical inhibition. Both conditions, however, are characterized by overall low contrast sensitivity, which can significantly influence collinear modulation.

Specifically, when the contrast of the flankers is similar to that of the target, facilitation can occur at 1.5–2λ instead of the classical inhibitory pattern typically observed at these short target-to-flanker separations. This observation aligns with findings by Contemori and colleagues^30^, who demonstrated that MD patients exhibited facilitation rather than inhibition at short separations under conditions where the target contrast approached that of the flankers.

Although structural or functional cortical differences cannot be entirely excluded, these observations suggest that altered collinear modulation patterns in MD are better explained by the influence of similar target and flanker contrasts, rather than by use-dependent cortical reorganization. As highlighted by Contemori et al. (2019), the facilitation observed in MD patients at short separations aligns with the contrast-dependent contextual effects described in the Zenger and Sagi^21^ model. This interpretation offers a unified framework for understanding collinear modulation across both healthy and clinical populations, reconciling inconsistencies in the existing literature. Further, our data at 4λ shows facilitation only at medium-high flankers contrast and no effect at very low flanker contrast. Figure 10b from^20^ seems to suggest that inhibition may occur even in a 4λ condition with very high flanker contrast. However, our data do not support this notion. In fact, we observed facilitation even when the flanker contrast was sixty time the value of the baseline.

To summarize foveal results:

- In healthy individuals, our data indicate opposing effects of flankers depending on target contrast: inhibition at high target contrast and facilitation at low target contrast.
- Psychophysical data demonstrate inhibition at 2λ or shorter distances with high flanker contrast and facilitation with low flanker contrast. At larger distances (4λ), only facilitation is observed.
- It is plausible that contextual modulation in clinical populations differs not due to structural or functional differences in visual areas but solely based on perceived flanker contrast.

Peripheral collinear modulation: In Experiment 3 we tested collinear facilitation in the near periphery, with the results painting a more complex picture. We observed predominantly positive TEs, regardless of flanker contrast. This indicates that the presence of flankers consistently led to inhibitory effects, with the strength of inhibition increasing as the contrast of the flankers increased at 3λ, consistent with prior studies. At 8λ, where previous research anticipated facilitation (i.e^11^. , , we observed inhibition instead, but only at low to intermediate levels of flanker contrast). It is possible that the inhibition observed in the periphery could be influenced by visual crowding effects, which may mask the typical facilitation provided by high-contrast collinear flankers. Additionally, the effect of attention on visual targets tends to be stronger in the peripheral visual field compared to the fovea^27^, suggesting that attentional shifts in peripheral vision might affect the results. In general, collinear modulation seems to be less robust among participants as the stimulus’ distance from fixation increases^27^. Unlike foveal collinear modulation where parameters leading to facilitation and inhibition are well-established, literature that tested this effect in the periphery reported more variable results^6,25–27,41^. However, even with these considerations, negative TEs were not observed, differing from findings reported in previous studies^6,25,27,41^.

A possible explanation for this apparent inconsistency may lie in methodological differences between previous studies and our approach. Unlike earlier studies measuring collinear facilitation in the periphery and presenting the triplet on only one side of the screen, we presented the flankers on both sides of fixation. This modification may have increased spatial uncertainty, as the flankers no longer provided a location cue for the target’s appearance. Indeed, previous research has linked collinear facilitation with reduced spatial uncertainty^28,43^. Overall, the results from the peripheral experiment appear at odds with evidence from clinical population with central vision loss, in which reduced inhibition at short separations was observed^17,30^. This suggests that the model of Zenger and Sagi may not hold true for individuals with low contrast sensitivity (CS) due to visual impairment, such as macular degeneration, thus offering indirect evidence for cortical plasticity effects in this population.

Limitations: Importantly, the formula for defining Gabor stimuli varies in the literature. For example, some researchers include a factor of 2 in the denominator of the exponential term, which is not used in our experiments. While our formula aligns with that of Zenger and Sagi^21^, it is crucial to be mindful of these differences when making comparisons between studies.

Another important consideration is the influence of practice effects: previous work^21,27^ documented changes in collinear facilitation/inhibition over multiple sessions. While we took steps to control short-term practice effects within each session, we cannot rule out that more extensive practice could alter the observed patterns, especially for peripheral viewing. Our foveal results replicated classical facilitation/inhibition effects with high contrast flankers, suggesting limited practice influence over a single session. However, the greater variability seen in our peripheral data aligns with previous reports of individual differences in peripheral collinear modulation that could potentially be mitigated by more extensive practice regimens. Future studies systematically tracking changes across multiple practice sessions are needed to fully characterize plasticity of lateral interactions in foveal and peripheral vision. Understanding practice dynamics is crucial for using collinear configurations in visual training/rehabilitation. Combining such studies with computational modeling may elucidate the experience-dependent neural mechanisms underlying contextual influences in perception. Elucidating practice effects represents an important next step towards a comprehensive account of collinear facilitation and inhibition.

In the peripheral configuration, we did not control fixation with an eye-tracker. Although this could be seen as a limitation, the short presentation duration and the randomization of target presentation between the left and right hemifields reduced the likelihood of participants making a saccade to view the target with the fovea instead of the periphery^44,45^. Additionally, since the target appeared randomly in either the left or right visual field, participants could only guess the target’s position correctly 50% of the time, making eye movements less advantageous.

Currently, there is broad agreement on the importance of conducting a power analysis prior to running experiments. Although we considered this issue before data collection, we had reservations due to the effect sizes reported in existing literature, which often involve very small sample sizes (sometimes fewer than five participants). We acknowledge that power analysis is not a panacea; it relies on accurate estimates of the expected effect size, data variability, and other study characteristics. Inaccurate estimates can undermine the validity of the power analysis. Given these considerations, we chose to collect data from 20 participants per experiment, exceeding the sample sizes reported in many previous studies, despite the associated effort and costs.

The effect of collinear modulation in the fovea with high flanker contrast has been replicated in several studies with very limited sample sizes of just a few observers. Given this previous work, we were confident in being able to replicate this effect with a small sample. However, to the best of our knowledge, only a few studies, with few participants investigated the effect of flanker contrasts^21,24,46^.

Considering the paucity of data on this important parameter from studies with adequate sample sizes, we aimed to provide a more comprehensive characterization. We systematically varied flanker contrasts across multiple levels, as well as other key factors like spatial frequency, target-flanker separation distances, and eccentricity. To err on the side of caution for the foveal experiments, we used a sample size of 20 participants, larger than most previous studies. For the peripheral experiments, there is even more uncertainty in the literature, with conflicting results across studies. While 20 observers are a relatively large sample for foveal experiments, we recognize it may still be insufficient to achieve adequate statistical power when studying more variable peripheral collinear effects. Nonetheless, our peripheral data are valuable for highlighting this variability, which is an important consideration - particularly for clinical applications where group differences may only be reliably detected with very large sample sizes.

## Conclusion

A better characterization of collinear modulation not only expands our understanding of how the visual system builds complex perception from the basic and local features processed by the early visual cortex, but it also holds significant promise for developing successful clinical interventions.

Indeed, our results, together with prior evidence, demonstrate that previous findings in amblyopia and macular degeneration, initially attributed to abnormal lateral interactions, can be reinterpreted within the framework of a healthy visual system by considering contrast of both flankers and target. Specifically, target and flankers having similar contrast, as may be seen in patients, result in facilitation and inhibition effects opposite to those observed in the ‘standard’ paradigm when the contrast of the flankers and target is very large.

The key takeaway from this study is that a thorough understanding of all factors contributing to collinear facilitation is essential for accurately interpreting plasticity effects, results in clinical populations, and training outcomes. Additionally, we emphasize rigor and reproducibility by characterizing these effects in a large number of participants and across several parameters and levels, unlike some of the foundational studies on these effects.

This study enhances our understanding of collinear facilitation in both the fovea and the periphery, shedding light on critical aspects of contextual modulation. In the fovea, our findings reinforce and extend previous research by demonstrating that collinear facilitation is influenced by the contrast of the flankers. This observation suggests that contrary to previous interpretations emphasizing structural or functional differences in visual processing areas, variations in collinear facilitation in amblyopic patients might instead be influenced by small difference in the contrast between the target and flankers. In the peripheral visual field, however, the situation is more complex. Our study did not consistently replicate the patterns of collinear modulation reported in earlier research. While our results confirm the presence of inhibition at high contrast levels, facilitation was not observed. These discrepancies underscore the variability in peripheral collinear modulation and suggest that additional factors, such as visual crowding and the role of attention, may influence the observed effects. Given these findings, it is evident that collinear modulation in the periphery exhibits a more variable and less predictable pattern compared to the fovea. This variability highlights the need for cautious interpretation of peripheral results and underscores the importance of considering a range of factors, including flanker contrast, distance from fixation, and individual differences. Our study emphasizes the necessity of accurately characterizing collinear modulation and understanding the influence of key parameters such as the contrast of the flankers in relationship with the contrast of the target. It is crucial that interpretations of collinear effects, particularly in clinical populations, account for these parameters to avoid misleading conclusions about plasticity and training outcomes. Future research should aim to further explore collinear modulation with refined experimental designs to elucidate the underlying mechanisms of these effects. Such efforts will improve our ability to apply these findings in clinical settings and advance our understanding of visual processing across different contexts. We believe that an updated framework can help guide the interpretation of effects in clinical populations and potentially inform translational applications in clinical rehabilitation.

## Electronic supplementary material

Below is the link to the electronic supplementary material.

Supplementary Material 1

## Data Availability

data is available on the Open Science Framework (OSF) at the link: https://osf.io/fepbt/?view_only=42d6cc1d2a3c43588adf3dc1fb6b9a97.
