# Supplementary material for: Characterization of contrast-mediated collinear interactions in the human visual system
Source: Sci Rep. 2025 Apr 7;15:11877. doi: 10.1038/s41598-025-94361-y (PMC11976974; doi:10.1038/s41598-025-94361-y)
Supplement: Supplementary file 1 — Supplementary Material 1 [file 41598_2025_94361_MOESM1_ESM.docx]

**Characterization of Contrast-mediated Collinear Interactions in the Human Visual System**

**Supplementary materials**

In this supplementary material, we plot our data using the same x and y axes as Figure 10b from the paper by Zenger & Sagi (1996). The models represented in the figures are polynomial regressions. This was achieved by calculating orthogonal polynomials for the SOA variable using the poly() function in R.


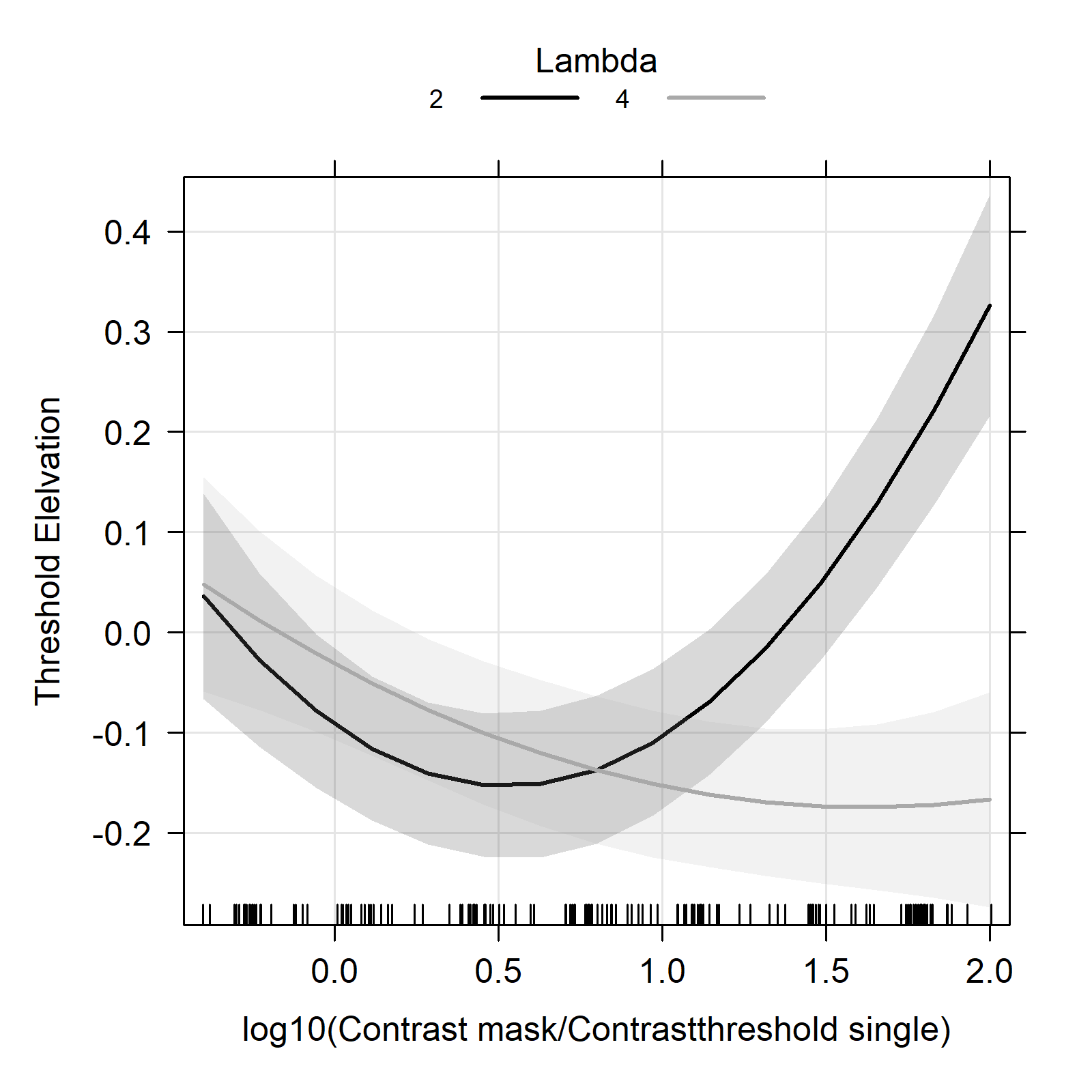


**Figure S1:** Data from Experiment 1 plotted using the x and y axes from Zenger and Sagi (1996).


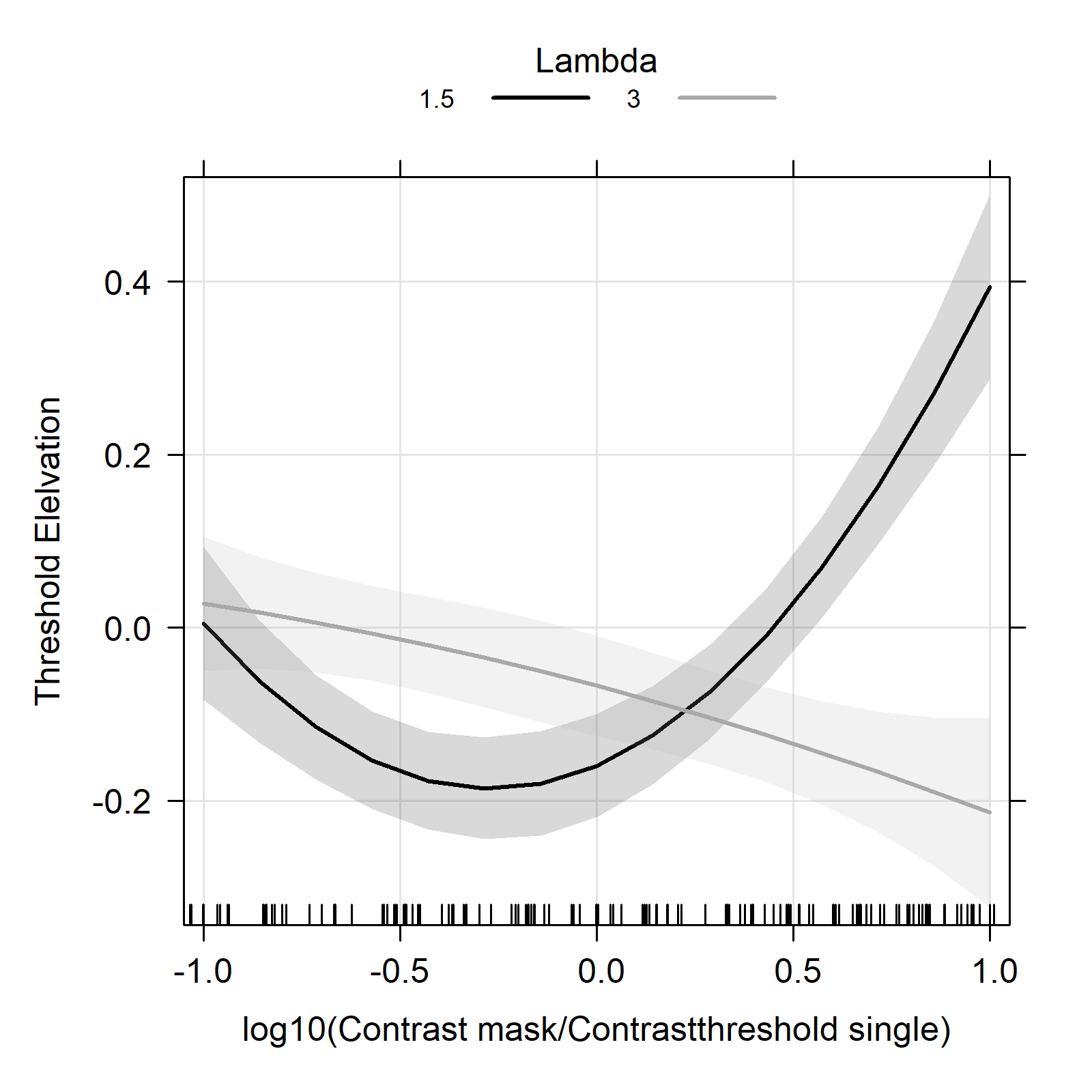


**Figure S2:** Data from Experiment 2 plotted using the x and y axes from Zenger and Sagi (1996).


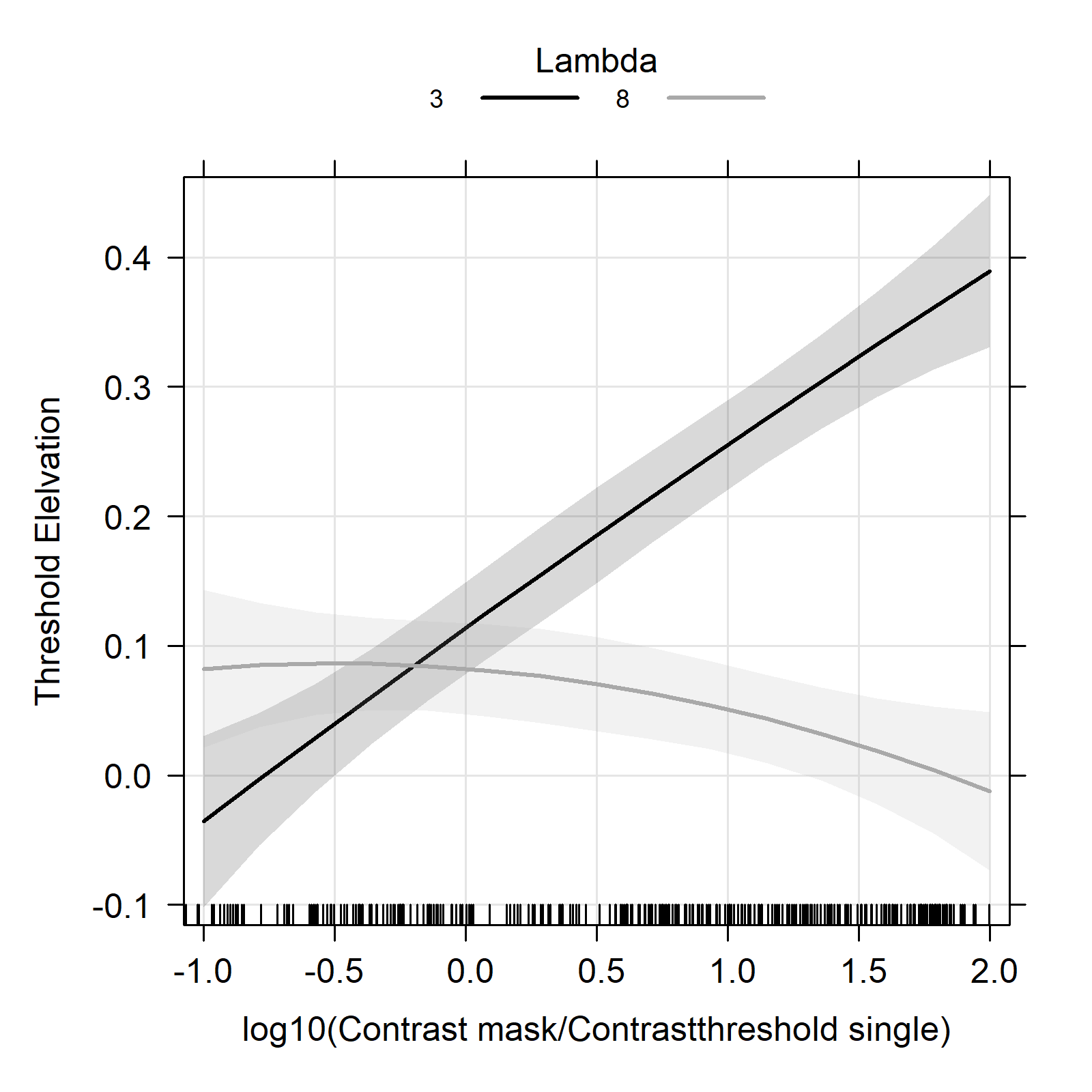


**Figure S3:** Data from Experiment 3 plotted using the x and y axes from Zenger and Sagi (1996).

In these figures, the x-axis was replaced with the formula from the Zenger & Sagi model (1996). The figures representing the results of Experiments 1 and 2 confirm the model, whereas this is not the case for Experiment 3 with the peripheral configuration (4°). These figures were created to compare empirical data with the mathematical model presented in Zenger & Sagi (1996).


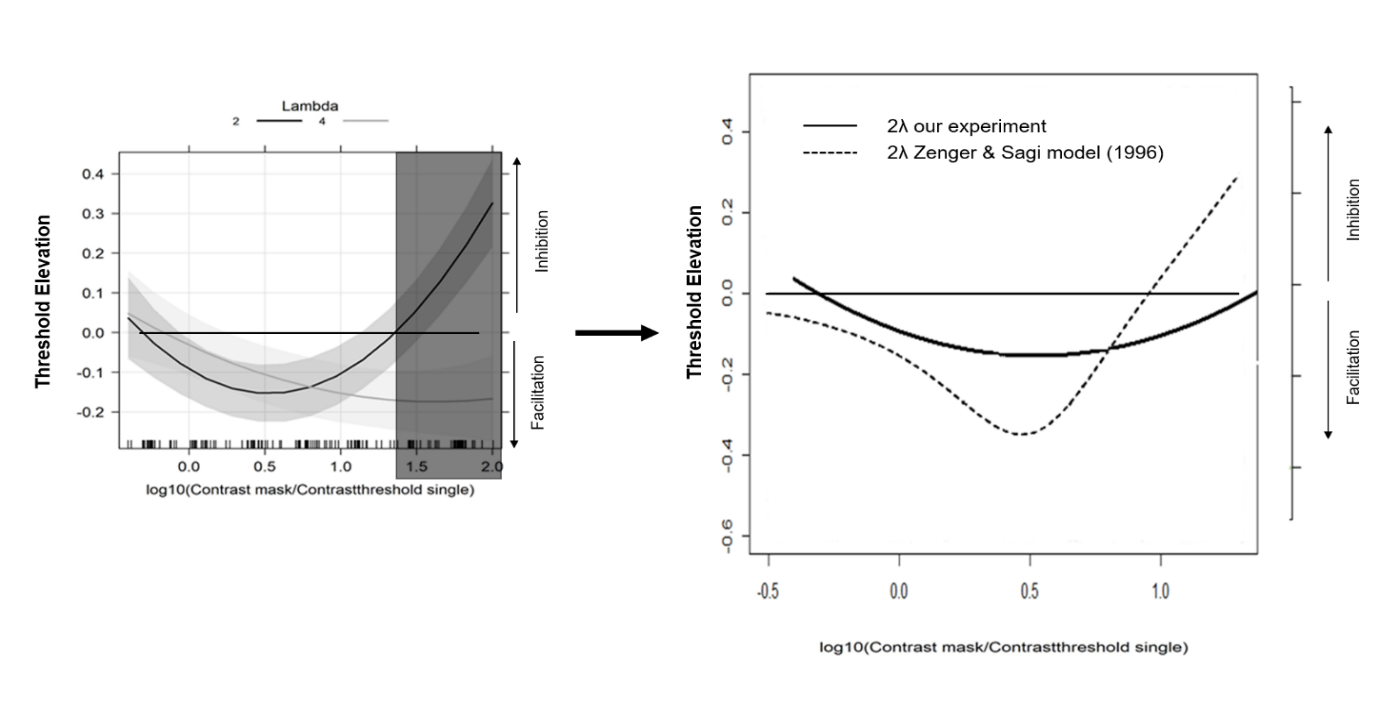


**Figure S4: Comparison between our data obtain in the 2lambda condition and data predicted by Zenger & Sagi model (1996)**

These graphs are designed to reveal the differences between empirical data and the mathematical model presented by Zenger & Sagi (1996). Although facilitation can be observed for low target-flankers ratios and low flanker contrast, our data show that facilitation is lesser than in the Zenger & Sagi model, and the transition point from facilitation to inhibition occurs later (at higher flanker contrast).
